# Angiopoietin-Like 8 in Gestational Diabetes Mellitus: Reduced Levels in Third Trimester Maternal Serum and Placenta, Increased Levels in Cord Blood Serum

**DOI:** 10.1155/2022/1113811

**Published:** 2022-04-26

**Authors:** Junhua Yuan, Di Zhang, Yunyang Wang, Zhen Zhu, Qian Lin, Manwen Li, Weizhen Zhong, Jing Han, Fengsen Xu, Jing Dong

**Affiliations:** ^1^Special Medicine Department, School of Basic Medicine, Qingdao University, Qingado, China; ^2^Shandong Provincial Engineering Laboratory of Novel Pharmaceutical Excipients, Sustained and Controlled Release Preparations, College of Medicine and Nursing, Dezhou University, Shandong, China; ^3^Department of Endocrinology & Metabolism, Affiliated Hospital of Qingdao University, Qingado, China; ^4^Department of Gynecology and Obstetrics, The First People's Hospital of Lianyungang, Lianyungang, China; ^5^Human functional laboratory, School of Basic Medicine, Qingdao University, Qingado, China; ^6^Department of Obstetrics, Qingdao Municipal Hospital, Qingdao University, Qingado, China; ^7^Department of Physiology, School of Basic Medicine, Qingdao University, Qingado, China

## Abstract

Gestational diabetes mellitus (GDM) poses a significant health risk to pregnant women, and thus exploring the potential underlying mechanism is highly desirable. The aim of the study was to compare maternal serum, cord blood serum, and placental angiopoietin-like 8 (ANGPTL8) levels in the third trimester of pregnancy in women with and without gestational diabetes and explore the potential underlying mechanism. A total of 42 pregnant women (23 with GDM and 19 with normal glucose tolerance (NGT)) along with 29 age-matched non-pregnant healthy females were enrolled. All pregnant subjects were in the late third trimester. Maternal serum and cord blood serum ANGPTL8 levels were measured with an enzyme-linked immunosorbent assay and the protein levels of ANGPTL8 in placentas were assessed with western blotting. The associations between maternal serum and cord blood serum ANGPTL8 levels and metabolic parameters were investigated with the Spearman correlation analysis. Significantly lower levels of maternal serum and placental ANGPTL8 levels were observed in GDM patients compared to NGT pregnant women, while remarkably higher ANGPTL8 levels were present in the cord blood serum samples. The maternal serum ANGPTL8 level was positively correlated with BMI, total cholesterol, triglycerides, and AUC for OGTT and birthweight. Additionally, the cord blood serum ANGPTL8 level was positively correlated with insulin and the homeostatic model assessment for insulin resistance. Both maternal serum and cord blood serum ANGPTL8 levels seemed to correlate with GDM and has the potential to be used as a biomarker for GDM and birthweight prediction.

## 1. Introduction

Gestational diabetes mellitus (GDM) is the most common complication in gestation, leading to macrosomia, fetal malformation, fetal growth restriction, abortion, and even fetal death *in utero* [1]. GDM is also associated with increased incidences of childhood obesity and hypertension in the offspring [2]. Increased insulin resistance (IR) during pregnancy is the main cause of GDM. Recently, Li et al. found that IR was associated with macrosomia in Chinese women with GDM [3]. Further mechanistic investigations suggested that hormones and adipocytokines secreted by placenta and adipose tissues are involved in the development of IR during pregnancy and may play important roles in the pathogenesis of GDM [4].

Angiopoietin-like 8 (ANGPTL8), a newly identified member of the angiopoietin-like protein family, is a 22-kDa peptide synthesized in the liver and adipose tissues [5]. ANGPTL8 is involved in glucose metabolism and plays a vital role in lipid metabolism. ANGPTL8 has been found to accelerate glycogen synthesis, improve glucose tolerance, and increase insulin sensitivity by promoting AKT/PKB phosphorylation [6]. It also regulates lipid metabolism by inhibiting the lipoprotein lipase (LPL) activity, thereby consequently increasing triglyceride (TG) levels and decreasing free fatty acid (FFA) levels in the serum [7].

Clinical studies have associated circulating ANGPTL8 levels with metabolic diseases such as diabetes and obesity [8]. Most studies have reported elevated levels of ANGPTL8 in diabetic patients and positive associations with IR-related indicators and TG levels [9, 10]. Currently, it is still controversial whether ANGPTL8 is involved in the management of glucose and lipid metabolism in GDM patients. By comparing ANGPTL8 levels among non-pregnant women, pregnant women with normal glucose tolerance (NGT) and GDM women, Huang et al. revealed significantly elevated ANGPTL8 levels in NGT pregnant women compared to non-pregnant women but no statistical difference was found between circulating ANGPTL8 levels in GDM pregnant women and normal pregnant women [11]. However, Erol et al. suggested that ANGPTL8 levels in GDM pregnant women were significantly higher than those of normal pregnant women [12]. The discrepancies may result from differences in the trimester of pregnancy, sample size, and various diagnostic criteria. Further investigation is urgently needed for better elucidation of ANGPTL8 level changes in GDM.

Although no obvious changes in lipid metabolism are present during early pregnancy, remarkably increased fat storage was observed during mid-pregnancy [13]. On the other hand, maternal IR was observed starting from the second trimester and peaks in the third trimester, in which rapid growth and development of the fetus requires high amount of energy and oxygen supply [14]. IR and relative hypoglycemia results in lipolysis, preserving the available glucose and amino acids for the fetus [15]. As described above, pregnancy is a diabetogenic state; if a woman is unable to mobilize pancreatic function to overcome the IR associated with pregnancy particularly at third trimester, then gestational diabetes would emerge [15, 16]. The placenta is a major endocrine organ, secreting more than 100 hormones that regulate maternal physiology [15]. Though ANGPTL8 was reported to play an important role in pregnant women with or without GDM, very little is known about ANGPTL8 levels in the late third trimester.

In the current study, the protein expression levels of ANGPTL8 in placenta of GDM and NGT pregnant women were assessed along with the maternal circulating ANGPTL8 levels, revealing the relationship between placenta ANGPTL8 and maternal ANGPTL8. Moreover, the correlation between maternal serum or cord blood serum ANGPTL8 and maternal glucose and lipid metabolic parameters were further analyzed in both groups to illustrate whether ANGPTL8 in maternal circulation and cord blood play the same role in modulation of maternal metabolism and to assess its potential to be used as a biomarker for GDM. Finally, we evaluated the consistency and feasibility of ANGPTL8 as a biomarker for GDM in complex clinical applications.

## 2. Patients and Methods

This prospective case-control study was conducted at Qingdao Municipal Hospital affiliated to Qingdao University, Qingdao, China, between December 2018 and May 2019. All the enrolled participants provided informed consent. The protocol was approved by the Institutional Research Human or Animal Ethics Committee of Qingdao Municipal Hospital (Approve code: 2018.39) and carried out in accordance with The Code of Ethics of the World Medical Association.

### 2.1. Patients

19 healthy pregnant women (age 29.21 ± 0.75 years, gestational age 240.16 ± 10.98 days) and 23 pregnant women (age 30.04 ± 0.76 years, gestational age 227.74 ± 9.08 days) with GDM were enrolled in the study, and 38 young women without pregnancy were selected as control subjects for the pregnant influence on ANGPTL8 levels. Echography was used for the determination of the accurate gestational age. Diagnosis of GDM was established by a 75 g oral glucose tolerance test (OGTT) after an overnight fast of 10 hours interpreted according to the criteria of American Diabetes Association [17]. NGT: fasting plasma glucose (FPG) < 5.6 mmol/l and 2-hour postprandial glucose (2 h FPG) < 7.8 mmol/l; GDM was diagnosed when any one of the following criteria were met during the OGTT: fasting > 5.1 mmol/l; 1 h > 10.0 mmol/l; 2 h > 8.5 mmol/l. Among GDM patients, nine had insulin therapy and fourteen were treated with diet alone. Maternal body mass index (BMI) was calculated as weight divided by squared height. Furthermore, the homeostasis model assessment (HOMA) was determined with the following formula: HOMA-IR = [fasting glucose (mmol/l) × fasting insulin (*μ*/ml)] /22.5. Exclusion criteria were as follows: type 1 or 2 diabetes before pregnancy, chronic hypertension, liver disease, active cancer, thyroid disorders, chronic renal failure on hemodialysis, fetal anomalies, multiple gestation, polyhydramnios, polycystic ovarian syndrome, history of smoking, chronic alcohol consumption, or chronic vascular disease.

### 2.2. Sample Collection

Fasting blood was collected from each patient prior to any interventions and were immediately centrifuged after clotting at 4°C. Cord blood serum and placental tissues were collected immediately after delivery. Cord blood was collected from the umbilical vein following standard protocols and then centrifuged at 4°C. Placenta tissues were obtained as previous described [18]. Briefly, the umbilical cord was cut after the delivery (remaining length is less than two centimeter), the fetal membranes were also removed. The placenta tissues were then rinsed free of blood with saline, dipped dry with a filter paper, and then tissue samples were collected from the central zone of placenta (1 x 1 x 1 centimeter). The supernatant serum and placenta tissues were kept in a −80°C freezer until further uses.

### 2.3. Laboratory Tests

The levels of plasma glucose, maternal serum total cholesterol (TC), TG, and insulin were measured with a biochemical analyzer (Beckman Coulter, Inc., Brea, CA, USA) at Qingdao Municipal Hospital affiliated to Qingdao University. Serum ANGPTL8 levels (both maternal and cord blood serum) were measured using a commercially available enzyme-linked immunosorbent assay (ELISA) (Wuhan Eiaab Science, Wuhan, China; Catalogue No. E11644h). Maternal serum FFA levels were determined using an ELISA kit (CLOUD CLONE CORP, Wuhan, China; Catalogue No. SEC971Hu) following the manufacturer's instructions. The protein expression levels of placental ANGPTL8 were determined with western blotting as previously described [19]. The primary antibody for ANGPTL8 was purchased from Phoenix Biotec (Beijing, China, WBH-051-55). The peroxidase-conjugated secondary antibody was purchased from ZS-BIO (Beijing, China). Bands were visualized with an Immobilon Western chemiluminescent substrate (Millipore, cat. no. WBKLS0100), and the intensities were analyzed with Image J software (NIH, US). Three independent blots from different samples were performed.

### 2.4. Statistical Analysis

Statistical analysis was performed using SPSS (Version 24.0; SPSS USA). The Shapiro–Wilk test was first performed to determine whether the data sets were normally distributed. When the data were normally distributed, data were presented as means ± SEM, and statistically significant differences among three groups were assessed by analysis of variance (one-way ANOVA) followed by post-hoc least significant difference tests. Differences between two groups were assessed with Student's *t*-test. When the data were not normally distributed, the Mann–Whitney *U* test was used to determine statistical significance, and data were presented as median (IQR). The correlation analysis was examined by two-way invariant correlations (normal distributed data: Pearson's correlation coefficient; non-normal distributed data: Spearman's correlation coefficient). The alpha level was set at 0.05.

## 3. Results

### 3.1. Levels of ANGPTL8 and Metabolic Dysfunctions in GDM

Clinical characteristics, levels of ANGPTL8, and the metabolic parameters of NGT and GDM patients are presented in Table 1. While no statistical differences were detected in terms of age (*P*=0.195), duration of gestational days (*P*=0.071), BMI (*P* = 0.872), FFA (*P*=0.752), and TC (*P*=0.146) levels between groups, the maternal serum ANGPTL8 levels were significantly elevated in NGT or GDM group compared with the control group (*P* < 0.05; Supplementary Figure 1). On the other hand, the levels of ANGPTL8 in patients with diabetes were lower than NGT pregnancy women (*P* < 0.05; Supplementary Figure 1). While compared with NGT, the ANGPTL8 levels of cord blood serum were significantly increased in the GDM group (*P* < 0.05; Table 1). Moreover, the glucose metabolism biochemical parameters were elevated significantly in GDM (*P* < 0.001) relative to the NGT group. Additionally, the HOMA-IR, AUC for OGTT, TG levels, and birthweight were all significantly increased in the GDM group comparing to NGT group (*P* < 0.001; Table 1, *P* < 0.001, Table 1, *P*=0.013, Table 1, *P*=0.002, Table 1, respectively).

### 3.2. Decreased ANGPTL8 Protein Expression Levels in Placenta with Increased ANGPTL8 Levels in Cord Blood Serum in GDM

**Figure 1:**
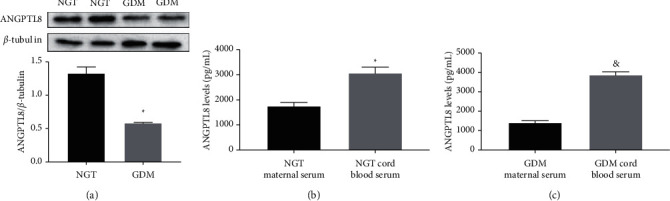
ANGPTL8 levels in maternal serum, cord blood serum and placenta. Maternal serum and cord blood serum samples were used to measure the ANGPTL8 levels in the samples. *N* = 19 for NGT pregnant women and *N* = 23 for GDM pregnant women. Placenta samples were assessed with western blotting for the ANGPTL8 expression levels in the samples. Three independent samples were used per group. (a) The protein expression level of ANGPTL8 in placenta. (b) Comparison of maternal serum and cord blood serum ANGPTL8 levels from pregnant subjects with normal glucose tolerance. (c) Comparison of maternal serum and cord blood serum ANGPTL8 levels from pregnant subjects with diabetes mellitus. ^*∗*^: statistically different from pregnant subjects with normal glucose tolerance (*P* < 0.05) &: statistically different from pregnant subjects with diabetes mellitus (*P* < 0.05).

Relative placental protein levels of ANGPTL8 were significantly decreased in samples from GDM group relative to those from NGT group (*P*=0.001; Figure 1(a)). Regarding to the cord blood serum, significantly higher ANGPTL8 levels were observed in both NGT and GDM groups relative to the corresponding maternal serum ANGPTL8 levels (*P* < 0.001, respectively; Figure 1(b), 1(c)).

### 3.3. Correlation between Maternal Serum ANGPTL8 and Metabolic Parameters

**Figure 2:**
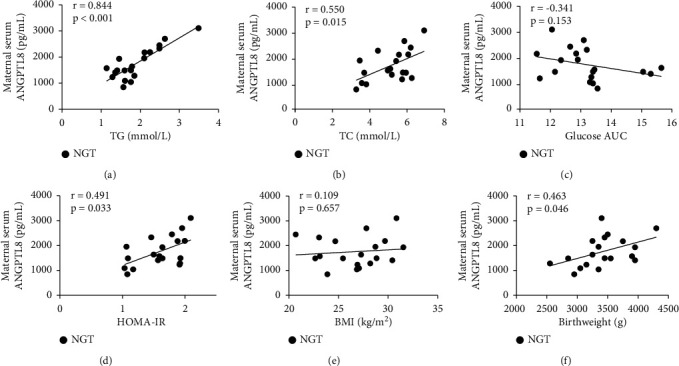
Correlation analysis between maternal serum ANGPTL8 and metabolic parameters of pregnant subjects with normal glucose tolerance. (a) Correlation between ANGPTL8 and TG levels. (b) Correlation between ANGPTL8 and TC levels. (c) Correlation between ANGPTL8 and AUC for OGTT. (d) Correlation between ANGPTL8 and HOMA-IR. (e) Correlation between ANGPTL8 and BMI. (f) Correlation between ANGPTL8 and birthweights.

**Figure 3:**
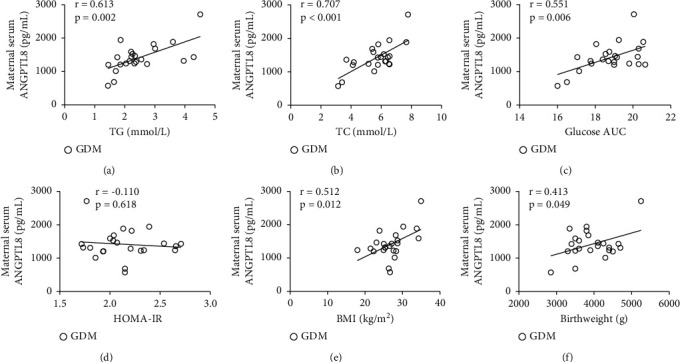
Correlation analysis between maternal serum ANGPTL8 and metabolic parameters of pregnant subjects with diabetes mellitus. (a) Correlation between ANGPTL8 and TG levels. (b) Correlation between ANGPTL8 and TC levels. (c) Correlation between ANGPTL8 and AUC for OGTT. (d) Correlation between ANGPTL8 and HOMA-IR. (e) Correlation between ANGPTL8 and BMI. (f) Correlation between ANGPTL8 and birthweights.

In the NGT group, circulating ANGPTL8 was positively associated with TG, TC, HOMA-IR, and birthweight (*P* < 0.05; Figure 2(a), 2(b), 2(d), 2(f)), while inversely associated with AUC for OGTT (*P* < 0.05; Figure 2(c)) and no correlation with BMI (*P* > 0.05; Figure 2(e)). In the GDM group, circulating ANGPTL8 was positively associated with TG, TC, AUC for OGTT, BMI, and birthweight, respectively (*P* < 0.05; Figure 3(a)–3(c), 3(e), 3(f)), while no correlation with HOMA-IR (*P* > 0.05; Figure 3(d)). No correlation was observed between ANGPTL8 and age, pregnant duration, FFA, FPG, and insulin levels both in NGT and GDM groups (Table 2).

### 3.4. Correlation between ANGPTL8 in Cord Blood Serum and Metabolic Parameters

Positive correlations were observed between ANGPTL8 and birthweight in both NGT and GDM group (*P* < 0.05; Table 3). However, inverse correlations were detected between ANGPTL8 and HOMA-IR or FPG in GDM group (*P* < 0.05; Table 3). No significant correlations were observed between ANGPTL8 and age, pregnant duration, BMI, FFA, TG, TC, insulin level, or glucose AUC both in NGT and GDM groups (*P* > 0.05; Table 3)

### 3.5. ANGPTL8 Levels Were Consistent in GDM Patients with Different Management

No significant differences were observed in ANGPTL8 levels between GDM patients received diet management or insulin treatment in cord blood serum (*P* > 0.05; Table 4).

## 4. Discussion

Although the role of ANGPTL8 in promoting pancreatic beta cell proliferation is controversial [20], it had been identified that ANGPTL8 plays important roles in both glucose and lipid metabolism [21]. Thus, ANGPTL8 could not be ignored as a potential object in the metabolic syndrome or endocrinology research. The current study focused on ANGPTL8 in pregnant women with or without metabolic disorders. The key finding was that significantly lower ANGPTL8 levels were observed in maternal serum and placenta tissues from GDM patients comparing to NGT objects. Positive correlations between maternal serum or cord blood serum ANGPTL8 levels and birthweight were also identified. Additionally, maternal serum ANGPTL8 levels were positively correlated to fatty acid metabolic parameters such as BMI, TC, and TG, while cord blood serum ANGPTL8 levels are primarily correlated with carbohydrate metabolic parameters such as FBG and HOMA-IR. Interestingly, no remarkable differences were observed in cord blood serum ANGPTL8 levels between GDM patients receiving diet management or insulin treatment.

### 4.1. ANGPTL8 in Maternal Circulation of Pregnant Women

Increased maternal serum ANGPTL8 levels were observed in pregnant women relative to non-pregnancy women, which was consistent with the findings of Trebotic et al. [22]. However, the current study showed a decreased level of ANGPTL8 in GDM group compared with NGT group, which was inconsistent with the results from Huang et al. [11] and Erol et al. [12]. The discrepancies may be explained by the differences in the time periods of pregnancy from which samples were collected and regional differences of people. Specifically, Huang et al. collected blood samples at gestational week 12–16, and Erol et al. collected blood samples at gestational week 25–26, while the current study collected blood samples at gestational week 28. The population difference (Turkish vs. Chinese) may also contribute to the differences observed in Erol et al. Interestingly, reduced placental ANGPTL8 protein expression was observed for the first time in GDM patients compared to NGT pregnant women. Since ANGPTL8 is known to reduce maternal serum TG levels [23] and improve glucose tolerance [22], the increased circulating ANGPTL8 levels in NGT objects may be a compensation to pregnancy-induced metabolic changes, while the GDM patients may have entered a decompensated state in ANGPTL8 synthesis. The placenta plays many important roles during pregnancy, including maintaining normal fetal physiology and protecting the fetus, as well as providing oxygen and nutrients for fetal development and growth [1]. Moreover, it is also worth noticing that the placenta, as an essential endocrine organ, secrets an extremely wide range of distinct peptide hormones into the maternal circulation [24]. Increasing emerged evidence suggested that some placental hormones participate in communications with maternal beta cells to maintain the glycometabolic adaptation for a healthy pregnancy [16]. The results from current study suggested that reduced maternal serum ANGPTL8 in GDM might be associated with the decreased production and secretion from placenta. As Wawrusiewicz-Kurylonek et al. did not observe a significant different of mRNA expression in placental tissue between GDM and NGT groups [25], post-transcriptional regulations are likely explanations for the observed protein level changes.

Dysfunction of metabolic parameters (TG, TC, insulin, HOMA-IR, FPG, OGTT, and glucose AUC) indicated remarkably increased metabolic burden in GDM patients, which coincides with relatively decreased circulating ANGPTL8 levels, suggesting that circulating ANGPTL8 is associated with the development of GDM. Further correlation analysis revealed a positive association between circulating ANGPTL8 levels and TC/TG levels in both GDM and NGT subjects, confirming previous reports of ANGPTL8-mediated LPL inhibition [7]. Moreover, positive associations with circulating ANGPTL8 were also detected for birth weight, which will be discussed later. Positive correlation was observed between IR and circulating ANGPTL8 in NGT subjects, while negative correlation was detected between AUC and circulating ANGPTL8, which are consistent with the fact that ANGPTL8 promotes beta cell activity thus improves glycometabolism [21]. In contrast, no significant correlations were observed between circulating ANGPTL8 and IR in GDM patients, while a positive correlation was observed between circulating ANGPTL8 and AUC, which may be the result of decompensated glycometabolism in GDM patients. In summary, maternal circulating ANGPTL8 levels may be utilized to predict the status of fatty acid metabolism.

### 4.2. ANGPTL8 in Cord Blood Serum and Placenta of Pregnant Women

Increased cord blood serum ANGPTL8 levels were observed in GDM relative to NGT pregnant women and higher than the corresponding maternal serum ANGPTL8 levels, which is consistent with previous reports [25, 26]. The opposite trends of cord blood serum and maternal serum circulating ANGPTL8 suggest differential roles of ANGPTL8 in maternal and fetal circulation. Cord blood serum ANGPTL8 was positively correlated with maternal 2 h OGTT, cord blood insulin, and HOMA-IR [26]. To the best of our knowledge, no direct reports stated the role of cord blood serum ANGPTL8 levels on other maternal glucose and lipid parameters. While the significance of this finding is yet to the elucidated, further investigation may be necessary for ANGPTL8 as a potential predictor of postdelivery maternal metabolic disorders. Interestingly, placental ANGPTL8 levels are not consistent with cord blood serum levels. While cord blood serum ANGPTL8 levels are higher in GDM patients, placental ANGPTL8 levels are lower in these patients comparing to normal pregnant women. Wawrusiewicz -Kurylonek et al. reported no significant changes in ANGPTL8 mRNA level in placental tissues [25]. Thus, the lower ANGPTL8 protein level observed in the current study might be the result of increased ANGPTL8 transportation from placenta to cord blood. However, no decisive explanations are available at this point. Further investigation is guaranteed.

### 4.3. ANGPTL8 Levels and Birthweights of Offspring

Trebotic et al. [27] reported a correlation between circulating ANGPTL8 and birthweight. The current study was consistent with their result and further revealed the relationship between cord blood serum ANGPTL8 level and birthweight. Cord blood serum ANGPTL8 also affected the outcome of the pregnancy, as it was positive correlated with birthweights of offspring both in GDM and NGT pregnant women. It suggested that maternal circulation and cord blood serum ANGPTL8 may not only function as a potential biomarker of maternal glucose and lipid dysfunction and metabolic status but may also serve as a predictor for GDM-related offspring problems, such as childhood overweight.

### 4.4. Consistency Evaluation of ANGPTL8 as a Biomarker in GDM Patients

The analysis for GDM patients is more challenging compared with NGT pregnant women, as in the late third trimester, GDM patients would be given some interventions according to their individual conditions. In the current study, the interventions given to GDM patients included insulin treatment and diet management. Our subgroup analysis of correlation suggested that even for patients who have been given different treatments, ANGPTL8 still has reference value as a biomarker.

### 4.5. Limitations of the Current Study

Several limitations of our study should be acknowledged. First, the sample size was limited and we assessed maternal serum ANGPTL8 levels only during the late third trimester of pregnancy without collecting the data from earlier trimesters, which may also contribute discrepancies compared to other studies. Second, although the study design was well suited to detect the robust correlations between ANGPTL8 and glucose and lipid metabolic parameters, no conclusions can be drawn regarding potential dynamic alterations during the course of gestation, as the parameters were only measured once during the pregnancy. However, the current results may instigate the investigation about the pathophysiologic relevance of this placental hormone, particularly in glycometabolic disorders during pregnancy. Additionally, the delivery type is not available in the current study, which may affect the ANGPTL8 levels. Moreover, future studies are needed to investigate whether the decreased production or secretion of ANGPTL8 from placenta could directly participate the development of GDM and macrosomia. Regarding the placenta expression results, ANGPTL8 is mainly synthesized in the liver and adipose tissue, and it is currently difficult to distinguish the contribution of placenta to fetus. It is well known that placenta exerts neural hormonal regulatory functions to the fetus [28, 29]; further investigation on placenta-synthesized ANGPTL8 is guaranteed.

## Figures and Tables

**Table 1:** Characteristics of pregnant subjects with normal glucose tolerance (NGT) and pregnant subjects with diabetes mellitus (GDM).

Variable	NGT (*n* = 19)	GDM (*n* = 23)	*P* Value
Age	28.00 (30.00–28.00)	30.00 (34.00–28.00)	0.195
Duration of pregnancy (day)	255.00 (268.00–225.00)	252.00 (252.00–217.00)	0.071
BMI (kg/m^2^)	26.72 ± 0.70	26.90 ± 0.84	0.872
FFA (ng/mL)	38.89 (75.59–22.67)	35.73 (60.35–22.44)	0.752
TC (mmol/L)	5.12 ± 0.25	5.66 ± 0.26	0.146
TG (mmol/L)	1.76 (2.25–1.46)	2.26 (2.96–1.85)	0.013^*∗*^
Insulin (uIU/mL)	7.00 ± 0.10	7.93 ± 0.15	<0.001^*∗*^
HOMA-IR	1.64 (1.92–1.17)	2.13 (2.34–1.93)	<0.001^*∗*^
OGTT-0h (mmol/L)	5.15 ± 0.14	6.08 ± 0.11	<0.001^*∗*^
OGTT-1h (mmol/L)	7.47 ± 0.17	11.40 ± 0.27	<0.001^*∗*^
OGTT-2h (mmol/L)	6.31 ± 0.17	8.68 ± 0.15	<0.001^*∗*^
Glucose AUC	13.21 ± 0.26	18.78 ± 0.27	<0.001^*∗*^
Birth weight (g)	3418.42 ± 97.94	3939.13 ± 116.85	0.002^*∗*^
Maternal serum ANGPTL8 (pg/mL)	1760.85 ± 139.13	1416.97 ± 89.97	0.046^*∗*^
Cord blood serum ANGPTL8 (pg/mL)	3077.81 ± 227.98	3883.1 ± 143.97	0.004^*∗*^

^*∗*^Statistically different from pregnant subjects with normal glucose tolerance (*P* < 0.05)

**Table 2:** Correlation analysis results between maternal serum ANGPTL8 and parameters of pregnant subjects with normal glucose tolerance (NGT) and with diabetes mellitus (GDM).

Variable	NGT (*n* = 19)		GDM (*n* = 23)	
	r	*P* Value	r	*P* Value
Age	−0.264	0.275	0.085	0.701
Duration of pregnancy (day)	0.054	0.828	−0.188	0.389
FFA (ng/mL)	0.375	0.113	−0.155	0.603
FPG (mmol/L)	−0.121	0.623	0.001	0.996
Insulin (uIU/mL)	0.119	0.627	−0.176	0.422

**Table 3:** Correlation analysis results between cord blood serum ANGPTL8 and parameters of pregnant subjects with normal glucose tolerance (NGT) and with diabetes mellitus (GDM).

Variable	NGT (*n* = 19)		GDM (*n* = 23)	
	r	*P* Value	r	*P* Value
Age	0.335	0.161	0.043	0.844
Duration of pregnancy (day)	0.226	0.353	0.280	0.195
BMI (kg/m^2^)	0.104	0.671	−0.031	0.887
FFA (ng/mL)	−0.047	0.847	0.014	0.950
TC (mmol/L)	−0.218	0.370	0.363	0.088
TG (mmol/L)	−0.119	0.626	0.030	0.893
Insulin (uIU/mL)	0.170	0.485	−0.214	0.326
HOMA-IR	−0.104	0.673	−0.438	0.037^*∗*^
FPG (mmol/L)	−0.391	0.098	−0.459	0.028^*∗*^
Glucose AUC	0.171	0.484	0.081	0.712
Birthweight (g)	0.587	0.008^*∗*^	0.533	0.009^*∗*^

^*∗*^Statistically different from pregnant subjects with normal glucose tolerance (*P* < 0.05)

**Table 4:** Treatment effects of diet management and insulin treatment in pregnant subjects with diabetes mellitus.

Variable	Diet management (*n* = 14)	Insulin treatment (*n* = 9)	*P* Value
Age	30.86 ± 1.07	30.33 ± 1.07	0.767
Duration of pregnancy (days)	251.00 (252.00–196.00)	252.00 (260.00–226.50)	0.305
Birth weight (g)	4100.00 (4425.00–3750.00)	3500.00 (4100.00–3375.00)	0.053
Cord blood serum ANGPTL8 (pg/ml)	3886.54 (4418.13–3633.66)	3901.49 (4350.68–3333.51)	0.926

## Data Availability

The underlying data supporting the results of the study are available from the corresponding author upon request.

## Supplementary Materials

Supplementary Materials Supplementary Figure 1: ANGPTL8 levels in maternal serum. ANGPTL8 levels in maternal serum from unpregnant healthy control group women (HC), pregnant women with normal glucose tolerance (NGT), or gestational diabetes mellitus (GDM) are presented. Maternal serum samples were collected from HC, NGT pregnant women, and GDM patients. Enzyme-linked immunosorbent assay (ELISA) was used to detect the ANGPTL8 levels in the samples. *N* = 29 for HC, *N* = 19 for NGT pregnant women and *N* = 23 for GDM patients. #: statistically different from HC (*P* < 0.05) ^*∗*^: statistically different from NGT pregnant women (*P* < 0.05).
