# Supplementary figures and images for: Angiopoietin-Like 8 in Gestational Diabetes Mellitus: Reduced Levels in Third Trimester Maternal Serum and Placenta, Increased Levels in Cord Blood Serum
Source: Int J Endocrinol. 2022 Apr 26;2022:1113811. doi: 10.1155/2022/1113811 (PMC9072024; doi:10.1155/2022/1113811)

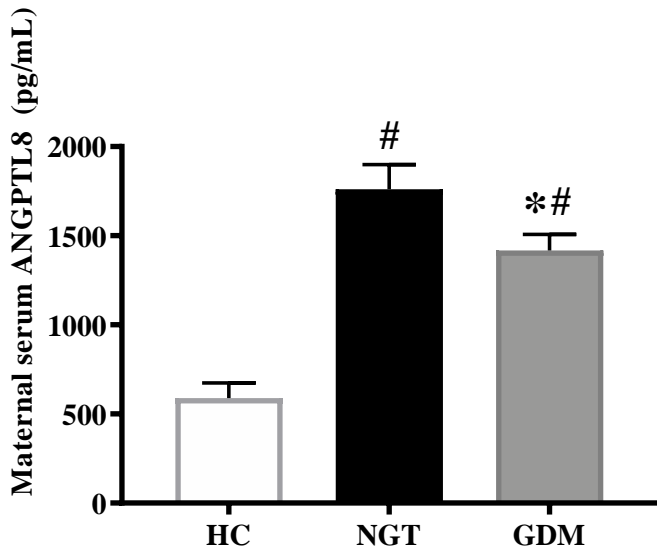

Supplement: Supplementary Materials — Supplementary Figure 1: ANGPTL8 levels in maternal serum. ANGPTL8 levels in maternal serum from unpregnant healthy control group women (HC), pregnant women with normal glucose tolerance (NGT), or gestational diabetes mellitus (GDM) are presented. Maternal serum samples were collected from HC, NGT pregnant women, and GDM patients. Enzyme-linked immunosorbent assay (ELISA) was used to detect the ANGPTL8 levels in the samples. N = 29 for HC, N = 19 for NGT pregnant women and N = 23 for GDM patients. #: statistically different from HC (P < 0.05) ∗: statistically different from NGT pregnant women (P < 0.05). [file 1113811.f1.pdf]
